# Characterization of a Novel Antisense RNA in the Major Pilin Locus of Neisseria meningitidis Influencing Antigenic Variation

**DOI:** 10.1128/JB.00082-15

**Published:** 2015-04-17

**Authors:** Felicia Y. Y. Tan, Mirka E. Wörmann, Edmund Loh, Christoph M. Tang, Rachel M. Exley

**Affiliations:** Sir William Dunn School of Pathology, University of Oxford, Oxford, United Kingdom

## Abstract

Expression of type four pili (Tfp) is essential for virulence in Neisseria meningitidis. Pili mediate adhesion, bacterial aggregation, and DNA uptake. In N. meningitidis, the major pilin subunit is encoded by the *pilE* gene. In some strains, PilE is subject to phase and antigenic variation, which can alter Tfp properties and together offer a possible mechanism of immune escape. Pilin expression and antigenic variation can be modulated in response to environmental cues; however, the precise mechanisms of such regulation remain unclear. We identified a promoter in the *pilE* locus, 3′ of the *pilE* coding sequence, on the antisense (AS) strand which is conserved in meningococci. We show that this promoter directs transcription of an AS RNA that is expressed during specific growth phases and in response to salt stress. Furthermore, we demonstrate that the transcript encompasses sequences complementary to the entire *pilE* coding sequence and 5′ untranslated region. AS RNAs can regulate the gene on the sense strand by altering transcript stability or translation. However, by using Northern blotting, quantitative reverse transcription-PCR (RT-PCR), and Western blotting, we found no significant AS RNA-dependent changes in *pilE* transcript or protein level. Instead, our data indicate that the AS RNA influences pilin antigenic variation. This work provides further insights into the complex regulation of pilin expression and variation in pathogenic Neisseria.

**IMPORTANCE** Pathogenic Neisseria spp. express type four pili (Tfp) which are important for adhesion, aggregation and transformation. Some strains of N. meningitidis are able to vary the sequence of the major subunit (PilE) of the Tfp. The mechanisms underlying this variation are not fully defined, but the process requires several noncoding elements that are found adjacent to the *pilE* gene. In this work, we identified a *cis-*encoded RNA antisense to *pilE* in N. meningitidis. By using Northern blotting and RT-PCR analysis, we found that the RNA is expressed in stationary phase or following salt stress. Our work also indicates that this RNA does not significantly affect *pilE* or pilin expression levels but instead appears to modulate pilin variation.

## INTRODUCTION

Neisseria meningitidis (meningococcus) is a Gram-negative bacterium that is frequently carried asymptomatically in the human upper respiratory tract. Occasionally, the meningococcus causes meningitis and septicemia, which can result in death or devastating sequelae. N. meningitidis has evolved a number of strategies to colonize the upper respiratory tract, including adhesion to epithelial cells and immune evasion. The expression of type four pili (Tfp) by this important human pathogen contributes to both colonization and disease (1).

In the meningococcus, Tfp are filamentous structures composed of thousands of subunits of the major pilin PilE, along with accessory pilins arranged in a helical configuration to form the pilus fiber (2). Pili are important for interactions with host cells (3). *In vitro*, strains lacking *pilE* have a reduced capacity to adhere to epithelial cells or explanted human airway organ culture (4–6). In addition, Tfp are essential for colonization of the vascular endothelium, where they promote bacterial survival by enabling microcolony formation and resistance to blood flow (7), as well as pathogenesis through signaling events that lead to inflammation and migration across the blood-brain barrier (8, 9). Finally, Tfp are also required for transformation in Neisseria spp. (10).

N. meningitidis expresses one of two distinct classes of Tfp which are distinguished by the sequence of PilE and the *pilE* expression locus (11, 12). Among disease-causing isolates, class II *pilE* genes are highly conserved, while class I pilins are subject to high-frequency phase and antigenic variation (Av), enabling the bacterium to modulate its pilin sequence (13). Pilin variation has consequences on host cell interactions (14, 15) and signaling (16) and may also influence the formation of bacterial aggregates and confer resistance to host immune responses (17). Meningococci expressing class I Tfp harbor a single pilin locus containing multiple copies of silent *pilS* sequences upstream of the *pilE* gene. The *pilS* cassettes lack predicted promoters, ribosome binding sites, and sequences encoding the N-terminal region of PilE necessary for pilus assembly. Pilin Av has been well studied in the related species Neisseria gonorrhoeae and arises through homologous recombination between *pilS* cassettes and the *pilE* coding sequence, an event known as gene conversion (18, 19). This process requires RecA (20) and RecQ and RecO (21) and is governed by additional genetic elements within the *pilE* locus. For example, a sequence that is predicted to form a guanine quartet (G4) structure is located 5′ to *pilE* and is essential for gene conversion (22), while a Sma/Cla repeat situated 3′ of *pilE* enhances the efficiency of this process (23). Furthermore, transcription of a G4-associated *cis*-acting small noncoding RNA is thought to be required for G4 formation and subsequent pilin antigenic variation (24). Thus, noncoding DNA and RNA sequences have important roles in pilin variation.

Despite its key role in virulence, regulation of pilin expression and variation in N. meningitidis is not well understood. The majority of pilin regulation studies have been performed in N. gonorrhoeae, but the two organisms differ in their host niches and may thus exhibit distinct expression profiles or responses to environmental changes. There is variation in *pilE* promoter sequences both between Neisseria species and also among different N. meningitidis strains (25). In N. meningitidis, a number of proteins participate in pilin regulation upon bacterial contact with host cells. CrgA, a transcriptional regulator induced following initial contact of bacteria with epithelial cells, downregulates the expression of *pilE* (26). NafA, a protein which is upregulated and surface expressed after adhesion of N. meningitidis to host cells, is proposed to control piliation at a posttranscriptional level by preventing pilus bundling (27). In addition, the RNA binding protein Hfq influences *pilE* expression in N. meningitidis in a strain-specific manner. For example, in strain H44/76, loss of Hfq leads to a reduction in PilE levels (28), while in MC58 loss of Hfq results in PilE upregulation (29). Hfq functions by facilitating the interaction of small noncoding RNAs (sRNAs) with target mRNAs (30), or it can facilitate antisense RNA pairing (31); therefore, this finding indicates that expression of *pilE* may involve regulatory RNAs.

Through genome analysis, we identified a putative promoter for a *cis*-encoded antisense (AS) RNA within the region 3′ of the *pilE* gene of N. meningitidis. The aim of this work was to investigate the function of this RNA. AS RNAs have been identified in numerous bacterial species, and their roles are largely to regulate the sense transcript at the level of transcription, mRNA stability, or translation (for reviews, see references 32 and 33). We therefore examined the role of RNA in modulating pilin expression in N. meningitidis. Moreover, given the importance of noncoding *pilE*-proximal elements in modulating pilin Av, we also sought to establish whether this RNA has any influence on this important process and found that expression of the RNA modulates pilin variation. Our work provides further insights into the complexity of the *pilE* locus.

## MATERIALS AND METHODS

### Strains, plasmids, and growth conditions.

Strains and plasmids used in this study are described in Table 1. Escherichia coli strains were grown on Luria-Bertani media at 37°C containing carbenicillin (100 μg/ml), kanamycin (50 μg/ml), or erythromycin (200 μg/ml) when required. N. meningitidis was grown overnight at 37°C, 5% CO_2_, either on brain heart infusion (BHI; Oxoid) agar supplemented with 5% heat-denatured horse serum and 0.1% starch or in liquid BHI. Erythromycin (2 μg/ml) was added for selection.

**TABLE 1:** Strains and plasmids used in this work

Strain or plasmid	Description and/or relevant property(ies)	Reference or source
E. coli DH5α	*fhuA2 lac*Δ*U169 phoA glnV44 *ϕ80Δ *lacZ*ΔM15* gyrA96 recA1 relA1 endA1 thi-1 hsdR17*	
N. meningitidis strains		
8013	Serogroup C, serotype 18, class I *pilE*	40
8013ΔG4	Kanamycin cassette inserted into G4 sequence	This study
WT_ery	Erythromycin cassette upstream of AS promoter	This study
Mut_ery	Erythromycin cassette upstream of AS promoter, AS promoter mutation	This study
Plasmids		
pGEM-T	Cloning and expression vector, carbenicillin resistance	Promega
pCR.2.ITOPOery	Cloning and expression vector, erythromycin resistance	R. Exley, unpublished data
pEGFP-N2	Cloning and expression vector, kanamycin resistance	Clontech
pEGFP-N2(Insert1)	N. meningitidis 8013 AS promoter region cloned into pEGFP-N2	This study
pEGFP-N2(Insert1_M1)	N. meningitidis 8013 AS promoter region cloned into pEGFP-N2; mutation of −35 site	This study
pEGFP-N2(Insert1_M2)	N. meningitidis 8013 AS promoter region cloned into pEGFP-N2; mutation of −10 site	This study
pEGFP-N2(Insert1_M3)	N. meningitidis 8013 AS promoter region cloned into pEGFP-N2; mutation of −35 and −10 sites	This study
pEGFP-N2 (AS_lpx_ery)	Vector for construction of WT_ery	This study
pEGFP-N2 (AS_lpx_ery_M3)	Vector for construction of Mut_ery	This study

For stress experiments, liquid cultures of E. coli or N. meningitidis were grown for approximately 3 h in LB or BHI medium, respectively, to an optical density at 600 nm (OD_600_) of 0.5 to 0.6 and subjected to acid stress (HCl, pH 2.5), envelope stress (5% Triton X-100 or 5% ethanol), oxidative stress (0.15% H_2_O_2_), salt stress (0.5 M NaCl or 0.5 M KCl), osmotic stress (6% sucrose), or temperature stress (10°C) for 10 min. For analysis of transcript and protein expression during growth, N. meningitidis strains were grown overnight on BHI agar. Bacteria were recovered from the plate, resuspended in phosphate-buffered saline (PBS), and quantified using the *A*_260_ to measure DNA in a lysed sample. A volume equivalent to 1 × 10^9^ CFU was inoculated into 25 ml of BHI medium in 125-ml conical flasks (Corning), and cultures were shaken at 180 rpm at 37°C. OD_600_ readings were taken at periodic intervals to determine growth phase, and samples were taken at various time points for RNA and protein analyses.

### Strain construction.

A 595-bp fragment, including the AS promoter (downstream), and a 752-bp adjacent fragment (upstream) were amplified from N. meningitidis 8013 using primers *pilE*_AS-F/*pilE*_AS-1R and lpxC_frag_F/R (Table 2), respectively. The PCR products were cloned flanking an erythromycin resistance cassette to obtain plasmid pEGFP-N2(AS_lpx_ery). Site-directed mutagenesis (QuikChange kit; Agilent) was carried out using primers SDM_mut3_F/R to obtain plasmid pEGFP-N2(AS_lpx_ery_M3). Plasmids were digested with HindIII and SacI, and the purified inserts were used to transform N. meningitidis 8013 to obtain the strains WT_ery and Mut_ery, respectively. Strain 8013ΔG4 was constructed by using fusion PCR to insert a kanamycin resistance cassette, including two *rrnB* terminators (34), into the guanine quartet region (CCCACCCAA^CCCACCC) so that the transcriptional orientation of the cassette was divergent to *pilE* (see Fig. S1 in the supplemental material). For the construction of strains WT_ery(RecA6) and Mut_ery(RecA6), genomic DNA from strain 8013 containing the *recA6* allele (gift from Hank Seifert) was used to transform the strains WT_ery and Mut_ery, respectively. Single colonies of the transformants obtained were confirmed by Western blotting of whole-cell extracts for RecA expression in the presence or absence of isopropyl-β-d-thiogalactopyranoside (IPTG).

**TABLE 2:** Oligonucleotides used in this study

Oligonucleotide purpose and name	Sequence	Purpose
Strain construction		
*pilE*_AS-F	GGGGGAATTCCGCGCCTGTCAGATAAACC	Amplification of *pilE* antisense; EcoRI site is underlined
*pilE*_AS-1R	GGGGCCCGGGCCGAAGCCATCCTTTTGGC	Amplification of *pilE* antisense; XmaI site is underlined
Ery_HindIII_F	GGGGAAGCTTCCGATACCCCCGATGACG	Amplification of Ery; HindIII site is underlined
Ery_SacI_R	GGGGGAGCTCGAATTCGCCCTTCCCGGGG	Amplification of Ery; SacI site is underlined
*lpxC*_fragment_F	GGGGGAGCTCCCCTGTCGCCGTCATTCC	Amplification of *lpxC* fragment; SacI site is underlined
*lpxC*_fragment_R	GGGGGCTAGCGCGTTCAGCTCAATCAGCG	Amplification of *lpxC* fragment; NheI site is underlined
SDM_mut3_F	CACTTACCGCT**CCC**TTTATTTAAAATTTATGGT**CAC**ATTTACCTTAGC	Site-directed mutagenesis of −35 and −10 sites of putative AS promoter; mutations are in bold
SDM_mut3_R	GCTAAGGTAAAT**GTG**ACCATAAATTTTAAATAAA**GGG**AGCGGTAAGTG	Site-directed mutagenesis of −35 and −10 sites of putative AS promoter; mutations are in bold
pEGFP_seq_F	GGTGGGAGGTCTATATAAGC	Sequencing of AS fragment in pEGFP-N2
insert1_seq_R	GGTTTATCTGACAGGCGCG	Sequencing upstream of AS fragment
GFP-seq-R	CGTCGCCGTCCAGCTCGACCAG	Sequencing of AS fragment in pEGFP-N2
*lpxC*_frag_R2	GGAAAAAATAGAAAGCGTTATCC	Amplification and sequencing of *lpxC* in N. meningitidis 8013
Ery_F_R	GCACGAGCTCAAGCTTCG	Sequencing of WT_ery and Mut_ery
Strand-specific qRT-PCR		
ssQRTPCR_tag_F	CCGTCTAGCTCTCTCTAATCG	Strand-specific RT-PCR (ssRT-PCR) tag
ssRTPCR_AS_tag	CCGTCTAGCTCTCTCTAATCCGTAAGCTTGAGGCATTTCC	cDNA synthesis for ssRT-PCR with AS; tag sequence is underlined
ssQRTPCR_AS_F	CGCCAAAATGCCGACGATG	ssRT-PCR with AS
ssRTPCR_*pilE*_tag	CCGTCTAGCTCTCTCTAATCGGATGGCTTCGGAAACTTGTG	cDNA synthesis for ssRT-PCR with *pilE*; tag sequence is underlined
ssQRTPCR_*pilE*_F	CGAGCTGATGATTGTGATTGC	ssRT-PCR with *pilE*
ssRTPCR_tmRNA_tag	CCGTCTAGCTCTCTCTAATCCCATTGACCGACTGCTGC	cDNA synthesis for ssRT-PCR with tmRNA; tag sequence is underlined
Northern blotting		
Insert1_probe	GCCGCCGCCAACGGCAAGACCGACGACAAAATCAACACCAAGCACCTGCC	Probe for AS transcript from pEGFP-N2(Insert1)
(AS)*pilE*-1	ACCGATGGTCAAATACATTGCATAATGCCGATGGCGTAAGCTTGAGGCAT	AS probe
*pilE*_probe	GGCTGATTTTTGACCTTCAGCCAAAAGGATGGCTTCGGAAACTTGTGCGC	*pilE* probe
8013_tmRNA_F	GGTTGCGAAGCAGATGCG	Amplification of N. meningitidis 8013 tmRNA PCR probe
8013_tmRNA_R	CCAGTCAATGTAAGATGACG	Amplification of N. meningitidis 8013 tmRNA PCR probe
ssrA_EC_F	CGAAACCCAAGGTGCATGC	Amplification of E. coli tmRNA probe
ssrA_EC_R	CAGGGCTTCCACGCG	Amplification of E. coli tmRNA probe
Antigenic variation assay primer *pilE*_F	CGATGGCGTAAGCTTGAGG	Amplification of *pilE*
Walking RT-PCR		
RT_F	AGCTGGCAGATGAATCATCG	Amplification of cDNA for mapping of 3′ end
RT_R1	CCCTTCAAAAAGGTTTTACCC	Amplification of cDNA for mapping of 3′ end
RT_R2	CAAACTTGATACCAATCTTGCT	Amplification of cDNA for mapping of 3′ end
RT_R3	CCATGCCAATAGAGATACCC	Amplification of cDNA for mapping of 3′ end
RT_3.5	TATGCTACCGCGCAAATTCAAA	Amplification of cDNA for mapping of 3′ end
RT_R4	CCAACCCACCCTATGCTAC	Amplification of cDNA for mapping of 3′ end
RT_4.5	CCAAGAAAACGGAAATTTTTAAAAA	Amplification of cDNA for mapping of 3′ end
T7_iv_temp_F	GGGGGAATTCTAATACGACTCACTATAGGAGCCTTGAAGCGCAGTCG	Amplification of template for *in vitro* transcription; T7 promoter is underlined
iv_temp_R	GCGGAGCGGTTTCTGTTGC	Amplification of template for *in vitro* transcription

### SDS-PAGE and Western blotting.

Whole-cell extracts were prepared from cultures at different time points by boiling bacterial suspensions in SDS-PAGE lysis buffer (35) at a dilution of 1 ml per OD_600_ unit. Proteins were separated by SDS-PAGE alongside molecular weight markers (All Blue; Bio-Rad) and transferred to nitrocellulose membranes (Hybond-C Extra; Amersham) for Western blotting. PilE was detected using SM1 (1:10,000) (36) and IRDye 800CW–goat anti-mouse IgG (1:10,000; Li-Cor). A GroEL loading control was detected using anti-GroEL antibody (1:8,000; gift from Jörgen Johansson) and IRDye 680–goat anti-rabbit IgG (1:10,000; Li-Cor). RecA was detected using an anti-RecA antibody (1:6,000; Abcam) and IRDye 680–goat anti-rabbit IgG (1:10,000; Li-Cor). Bands were visualized and quantified using the Odyssey Sa infrared imaging system. Experiments were carried out in triplicate using strains from independent transformations. PilE band intensities were normalized to the respective GroEL band intensities and expressed as the ratio to the normalized PilE value of the first lane.

### RNA isolation and Northern blotting.

Total RNA was isolated from E. coli by using the Fast RNA Blue kit (MP Biomedicals) followed by DNase treatment (Roche) or from N. meningitidis by using the RNeasy midikit and on-column DNase treatment (Qiagen). RNA (20 μg) was separated by electrophoresis in 1.5% agarose formaldehyde gels in HEPES buffer, transferred onto Hybond-N membranes (GE Healthcare), and cross-linked by UV light. Probes were generated by end-labeling primers [*pilE*_probe, (AS)*pilE*-1, or Insert1 probe], or a PCR product generated using primers 8013_tmRNA_F/R with [γ-^32^P]ATP (PerkinElmer). Membranes were incubated in hybridization buffer (GE Healthcare) and hybridized with labeled probes overnight at 64°C. Signals were detected using a fluorescent image analyzer (Fuji FLA-5000) and quantified using AIDA image analyzer software. Experiments were carried out in triplicate using strains from independent transformations.

### Strand-specific quantitative reverse transcription-PCR (qRT-PCR).

First-strand cDNA was synthesized from 5 μg total RNA by using AS- or *pilE*-specific primers carrying a 5′ tag sequence not found in the N. meningitidis genome (Table 2). RNAs were reverse transcribed in the presence of actinomycin to avoid second-strand cDNA synthesis. After RNase H treatment for 20 min at 37°C, cDNA was purified, and quantitative real-time PCR was performed with Power SYBR green PCR master mix (Applied Biosystems) using a unique tag primer, to ensure strand-specific cDNA amplification. The reaction was monitored using a StepOnePlus real time PCR system. Results are reported as the average *R* value (calculated as 2^−ΔΔ^^*CT*^, where *C_T_* is the threshold cycle) of triplicate experiments performed on WT_ery and Mut_ery strains obtained from three independent transformations. The amount of transcript was expressed as the *R* value standardized to the transfer-messenger RNA (tmRNA) control. The efficiencies of each primer pair were evaluated by performing a 10-fold dilution series experiment and determining the slope of the standard curves obtained. The primer pairs were found to have efficiencies ranging from 85 to 100%.

### Primer extension and walking RT-PCR.

RNA (10 μg) extracted from N. meningitidis strains following NaCl stress was mixed with labeled probe and denatured at 80°C for 5 min, and the primer was extended by using avian myeloblastosis virus reverse transcriptase in extension buffer (25 mM Tris-HCl [pH 8.3], 30 mM NaCl, 15 mM MgCl_2_, 1.25 mM dithiothreitol, 1 mM deoxynucleoside triphosphates) at 42°C for 1 h. Products were denatured at 80°C for 3 min in formamide sample buffer. A PCR product of the AS promoter region was generated using primers *pilE*_AS-F and *pilE*_AS-1R, and sequencing was performed using the labeled *pilE*_AS-1R primer according to standard methods (35). Products were run on a 5% denaturing polyacrylamide gel. Signals were detected as described previously.

Mapping (walking) PCR was performed according to published protocols (37). Total RNA was used as a template for the synthesis of separate cDNAs using primers R1 to R4.5 (Table 2). Reverse transcription was carried out using SuperScript III reverse transcriptase (Invitrogen) according to the manufacturer's directions. The respective cDNA primers and an upstream primer (AS_RT_F) were used for PCR amplification, and the PCR products were analyzed by agarose gel electrophoresis with a 1-kb DNA ladder (Bioline) for molecular size marker. Identical RT-PCR analysis was carried out on an *in vitro*-transcribed AS RNA synthesized with primers T7_iv_temp_F and iv_temp_R using the RiboMAX large-scale RNA production system (T7; Promega).

### Antigenic variation assays.

Antigenic variation assays in liquid medium were performed with strains 8013 and 8013ΔG4 or with WT_ery and Mut_ery strains containing the *recA6* allele. Each Av assay was carried out using bacteria inoculated from frozen stocks made from single colonies of the respective strains, in order to ensure that each experiment started with a homogenous population of cells containing the same *pilE* sequence. Strains grown overnight on solid BHI medium without IPTG were used to inoculate BHI broth and grown to mid-log phase. Where relevant, IPTG was then added to a final concentration of 1 mM and the cultures were incubated with shaking at 37°C for 10 min. For NaCl stress, the cultures were incubated with NaCl (0.5 M) together with IPTG for 10 min. The cultures were then serially diluted and plated onto BHI agar containing 1 mM IPTG and grown overnight for 22 h (see Fig. S2 in the supplemental material). Ninety-six colonies of each strain under each condition were passaged onto BHI agar and incubated overnight. Lysates were prepared by resuspending single colonies in 100 μl PBS and boiling for 10 min. *pilE* was amplified from single-colony lysates using primers AS_F and *pilE*_F, which are specific for amplification of *pilE*, and the Herculase II fusion DNA polymerase (Agilent). Products were sequenced and compared to *pilE* progenitor sequence, and changes were mapped to *pilS* donors. Each variant *pilE* was considered to have resulted from a single Av event. The assays with 8013 and 8013ΔG4 were performed once. Av assays with WT_ery(RecA6) and Mut_ery(RecA6) were performed three times.

### Statistical analysis.

The Student *t* test was used for the quantification of transcript level by strand-specific qRT-PCR. Fisher's exact test was used for the antigenic variation assay with strains 8013 and 8013ΔG4. Results of the antigenic variation assay with Wt_ery(RecA6) and Mut_ery(RecA6) were analyzed by fitting generalized linear models (GLMs) (38). Binomial GLMs were fitted, with the response variable defined as a sequence of ordered pairs (number of the variant, number of the same) from *pilE* sequences, and the predictor variables were the following: strain (WT_ery or Mut_ery), NaCl induction (yes or no), and assay (replicate 1, 2, or 3). R statistical software was used, specifically, the function GLM (39).

## RESULTS

### Identification of a putative promoter in the class I *pilE* locus in N. meningitidis.

**FIG 1:**
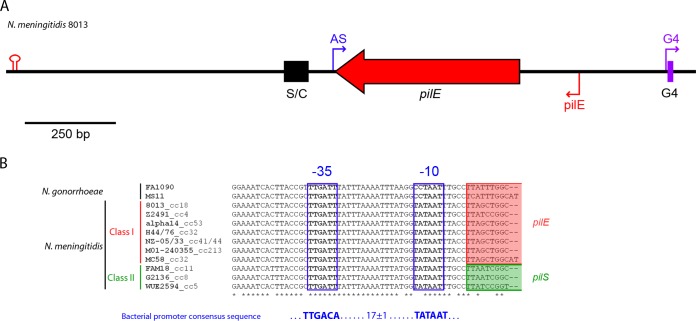
Location of a promoter sequence in the class I *pilE* locus in N. meningitidis 8013. (A) Schematic diagram of the pilin locus in N. meningitidis 8013. Sma/Cla (S/C) and guanine quartet (G4) are DNA elements in the *pilE* locus that are important for pilin antigenic variation in N. meningitidis. The promoters of the *pilE* AS RNA, the G4-associated sRNA, and *pilE* are denoted by the blue, purple, and red arrows, respectively. The predicted *pilE* terminator is denoted by the red stem-loop. (B) Multiple-sequence alignment of the region corresponding to the AS promoter in N. meningitidis strains from different clonal complexes (cc) expressing class I or class II pilin and that in N. gonorrhoeae. The putative −10 and −35 sequences are boxed in blue, *pilE* sequences are shaded in red, and *pilS* sequences are shaded in green. The promoter sequence is conserved in N. meningitidis strains. In the two strains of N. gonorrhoeae, the sequence is conserved, apart from three substitutions.

Inspection of the available genome sequence of N. meningitidis strain 8013 (40) (accession number FM999788) revealed the presence of a putative promoter on the AS strand, 5 nucleotides (nt) downstream of the *pilE* stop codon (Fig. 1A). No other putative promoters were identified on the AS strand within *pilE* in this sequence. The promoter has strong resemblance to the bacterial RpoD σ^70^ promoter consensus sequence and is conserved in meningococcal genomes among strains belonging to different lineages (Fig. 1B). In two N. gonorrhoeae genomes, the sequence is conserved apart from three substitutions, two of which occur in the putative −10 TATA box sequence (CCTAAT instead of TATAAT) (Fig. 1B). Interestingly, in meningococci that express class II pilin, the promoter is found in the *pilS* region but not adjacent to the *pilE* gene (Fig. 1B).

We did not identify any sequences resembling a Shine-Dalgarno ribosome binding site within proximity to an ATG start codon within 350 nucleotides of this promoter. We therefore hypothesized that this promoter drives transcription of a noncoding, AS RNA.

### Expression of the AS transcript in E. coli and N. meningitidis.

**FIG 2:**
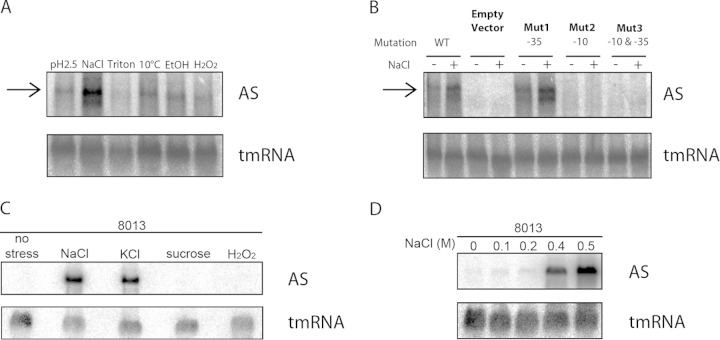
The AS transcript is induced by salt stress in E. coli and N. meningitidis. (A) A 595-bp region, including the AS promoter from N. meningitidis 8013, was cloned into pEGFP-N2 and transformed into E. coli; transformants were subjected to different stresses to identify conditions under which the AS promoter is functional. Northern blot analysis of total RNA was performed using a probe complementary to the cloned insert (AS) or transfer mRNA (tmRNA) as a loading control. The AS transcript was upregulated following NaCl stress. (B) Northern blot analysis of total RNA from E. coli strains containing plasmids with mutations to the −35 site (Mut1), the −10 site (Mut2), or both (Mut3) of the N. meningitidis *pilE* AS promoter with (+) and without (−) NaCl stress. No transcript was detected upon mutation of the −10 sequence. (C) Northern blot analysis of total RNA from wild-type N. meningitidis strain 8013 subjected to different stresses. The AS transcript was detected using a 50-nt probe [(AS)*pilE*-1] that hybridizes specifically to the AS RNA 533 nt downstream of the AS promoter. tmRNA was used as a loading control. A transcript corresponding to the AS RNA was detected following exposure to NaCl and KCl. (D) Analysis of AS RNA expression in N. meningitidis following a 10-min incubation with different concentrations of NaCl. AS RNA levels were increased at higher salt concentrations (0.4 and 0.5 M NaCl).

To determine whether the AS promoter is functional, we first examined the ability of this sequence to mediate transcription in E. coli. A 595-bp region from N. meningitidis, including the putative AS promoter and 386 bp of the 3′ end of the *pilE* coding sequence (but excluding the *pilE* promoter sequence) was introduced into pEGFP-N2 to make pEGFP-N2(insert1); this plasmid only harbors a cytomegalovirus early-immediate promoter for transcription in eukaryotic but not prokaryotic cells. To examine promoter activity, E. coli strain DH5α was transformed with pEGFP-N2(insert1), grown for 3 h to an OD_600_ of 0.5 to 0.6 in liquid medium, and then subjected to acid, NaCl, temperature, envelope, or oxidative stress for 10 min. The presence of the AS transcript was detected by Northern blotting by using a 50-nt probe that hybridized 39 nt downstream of the predicted −10 sequence. The AS transcript was significantly more abundant when the E. coli cells were exposed to high NaCl concentrations (0.5 M for 10 min) but not after other stresses (Fig. 2A). In addition, we generated plasmids in which the predicted −35 sequence (TTGATT → TCCCTT; Mut1), the −10 sequence (TATAAT → TCACAT; Mut2), or both (Mut3) had been modified in an attempt to abolish promoter activity (Fig. 2B). No transcript was detected in E. coli harboring pEGFP-N2 alone (empty vector) with or without salt stress. A transcript of the expected size was detected in E. coli containing the plasmid with the unmodified promoter sequence (wild type [WT]) or with an alteration in the putative −35 sequence (Mut1), indicating that the promoter is functional and that the −35 sequence is dispensable for expression of the RNA. In contrast, mutation of the −10 sequence (in Mut 2 and Mut 3) was sufficient to abolish transcription.

We also examined the expression of the AS RNA in response to different stresses in wild-type N. meningitidis. Strain 8013 was grown overnight on solid medium and used to inoculate liquid BHI medium. After 3 h growth at 37°C with agitation, bacteria were subject to stress conditions by addition of NaCl or KCl to 0.5 M, sucrose to 6%, or H_2_O_2_ to 0.15% for 10 min. Using a probe specific for the AS RNA, a single transcript was detected in bacteria treated with NaCl or KCl, indicating that the AS RNA is also expressed in response to salt stress in wild-type N. meningitidis (Fig. 2C). We subsequently determined the concentration of salt necessary to induce abundant expression of the AS RNA. Strain 8013 was grown in liquid medium as described above, and NaCl was added to a range of final concentrations from 0 to 0.5 M. As shown in Fig. 2D, AS RNA levels were increased at salt concentrations of 0.4 M or more.

**FIG 3:**
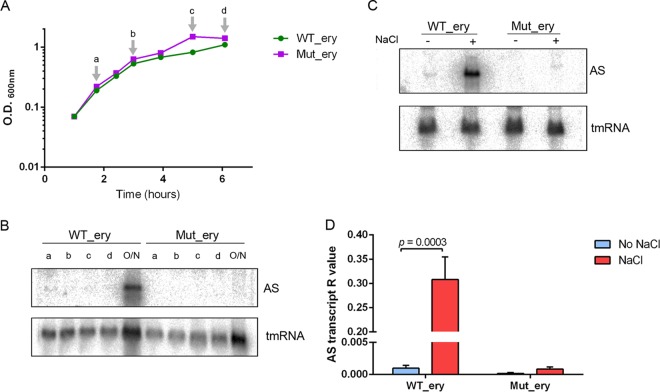
Expression of the AS transcript in N. meningitidis. (A) Growth curves of WT_ery and Mut_ery cultured at 37°C in BHI broth. Arrows indicate times at which RNA was extracted for Northen blot analysis of AS RNA expression. (B) Northern blot analysis results for total RNA isolated at different time points during growth (h 1.75 [a], h 3 [b], h 5 [c], h 6 [d], and after growth overnight [O/N] for 21.5 h). The AS transcript was detected using a 50-nt probe [(AS)*pilE*-1] that hybridizes specifically to the AS RNA 533 nt downstream of the AS promoter. tmRNA was used as a loading control. (C) Representative Northern blot analysis of total RNA from N. meningitidis strains WT_ery and Mut_ery, cultured with and without NaCl stress. (D) Strand-specific qRT-PCR analysis of AS transcript levels in WT_ery and Mut_ery strains grown with and without NaCl stress. Amount of transcript is expressed as the *R* value standardized to that of the tmRNA control. *P* = 0.0003, Student's *t* test. The AS transcript was upregulated under NaCl stress in WT_ery; no transcript was detected in Mut_ery.

To investigate the role(s) of the AS RNA, we next constructed isogenic strains with either an intact or a mutated AS promoter. The control strain of 8013, designated WT_ery, carries the erythromycin antibiotic resistance marker but an intact AS promoter sequence (see Fig. S3 in the supplemental material). We confirmed that AS expression in WT_ery was not affected by the presence of the erythromycin resistance cassette, based on Northern blotting (see Fig. S3). We also introduced the −10 and −35 AS promoter mutations into N. meningitidis strain 8013 by homologous recombination to generate the isogenic strain Mut_ery (see Fig. S3). There was no difference in growth of WT_ery and Mut_ery (Fig. 3A), and Northern blot analysis of total RNA isolated at different time points demonstrated that the AS RNA was detected in the WT_ery strain but not in Mut_ery after overnight growth (21 h, OD_600_ of 1.9) (Fig. 3B). In agreement with results using wild-type strain 8013, exposure of WT_ery to high salt (0.5 M NaCl) significantly increased the abundance of the AS transcript (by approximately 280-fold; *P* = 0.0003) (Fig. 3D; see also Table S1 in the supplemental material). Therefore, we have identified a *cis*-encoded AS RNA in the class I *pilE* locus of N. meningitidis strain 8013 which is present in stationary phase and can be upregulated by NaCl. In subsequent experiments, we chose to use 0.5 M NaCl stress as a method of inducing overexpression of the AS RNA to allow us to investigate its function.

### The AS RNA encompasses sequences complementary to *pilE* and its 5′ UTR.

**FIG 4:**
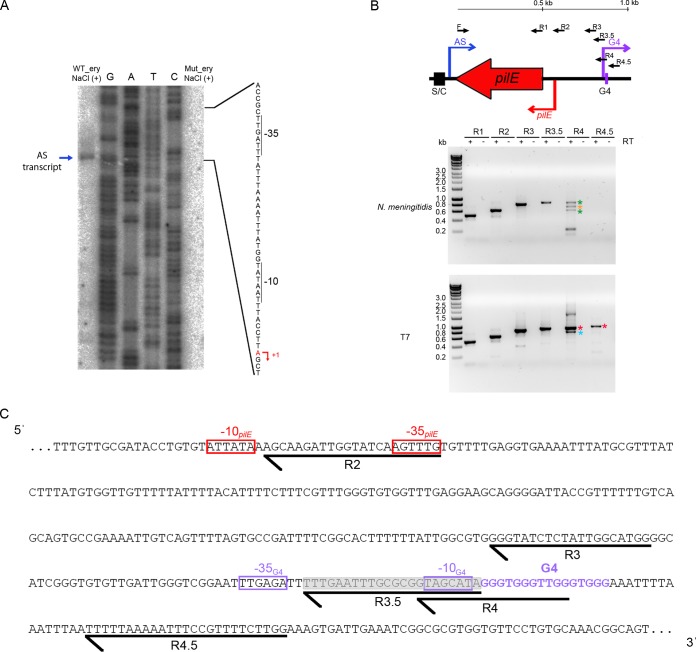
Characterization of the 5′ and 3′ ends of the AS transcript. (A) Primer extension analysis identified the transcriptional start site of the AS RNA in WT_ery following salt stress. The −10 and −35 sequences are indicated, and the transcriptional start site is shown (red arrow). No product was obtained using RNA from Mut_ery. (B, top panel) Mapping of the AS 3′ end by RT-PCR. Black arrows correspond to the hybridization positions of primers F and R1 to R4.5 used for PCR. The scale bar indicates the distance from the AS transcriptional start site. (Bottom panels) Agarose gel analysis of PCR products amplified using cDNAs from N. meningitidis WT_ery total RNA (upper panel) or *in vitro* T7-synthesized AS RNA (used as a template) (lower panel). The presence (+) or absence (−) of reverse transcriptase is indicated. A full-size 888-nt product obtained from amplification with primers F and R4 was detected using *in vitro*-synthesized RNA (red asterisks) but not total RNA from N. meningitidis, indicating that the *in vivo* transcript terminates before or within the sequence corresponding to primer R4. Green asterisks indicate nonspecific products amplified from rRNA, and orange and blue asterisks indicate truncated PCR products ascertained by sequencing. (C) Sequence of the region at the 3′ end of the AS RNA and location of primers R2 to R4.5. The G4 sequence is indicated in purple. The promoters of *pilE* and the G4-associated sRNA are depicted in red and purple boxes, respectively. The AS RNA is predicted to terminate within the shaded region.

AS RNA transcription can affect levels of the corresponding sense transcript through a variety of mechanisms, including altered RNA stability or binding to the 5′ untranslated regions (UTRs) of the sense mRNA to alter ribosome binding and thus affect protein expression. To determine whether the AS RNA encompasses the region complementary to the full *pilE* coding sequence and/or 5′ UTR, we mapped the 5′ and 3′ ends of the transcript. The 5′ end of the AS transcript was mapped by primer extension and found to be to 8 nt downstream of the −10 sequence of the predicted promoter. No extension product was obtained from the strain Mut_ery (Fig. 4A). RT-PCR was employed to map the 3′ end of the transcript using a set of reverse primers which hybridize at various intervals within the region corresponding to the *pilE* promoter and G4 sequence (Fig. 4B and C). As a control, the same PCR was performed using an AS RNA synthesized *in vitro* with T7 RNA polymerase. Results indicated that using RNA isolated from N. meningitidis as the template, a PCR product of expected size was obtained with primers F+R3.5 (877 nt) but not with F+R4 (888 nt) or F+R4.5 (933 nt). Products of 888 nt and 933 nt were amplified from *in vitro*-synthesized RNA with primers F+R4 and F+R4.5, respectively (Fig. 4B, red asterisks), demonstrating that the absence of product was not due to primer inefficacy. Smaller-sized products obtained from reactions using primers F+R4 were sequenced. The products obtained from N. meningitidis RNA corresponded to nonspecific amplification from rRNA (Fig. 4B, green asterisks) and a truncated PCR product of 727 nt and 733 nt (Fig. 4B, orange asterisk and blue asterisk, respectively). This result indicated that the 3′ end of the AS transcript in N. meningitidis lies between the site of hybridization of primers R3.5 and R4, which corresponds to the promoter of the G4-associated sRNA (Fig. 4C). Thus, the AS transcript encompasses sequence antisense to the entire *pilE* coding sequence and 5′ UTR and terminates within the promoter of the G4-associated sRNA (Fig. 4C, shaded region).

### AS RNA expression does not impact *pilE* transcript and pilin levels.

**FIG 5:**
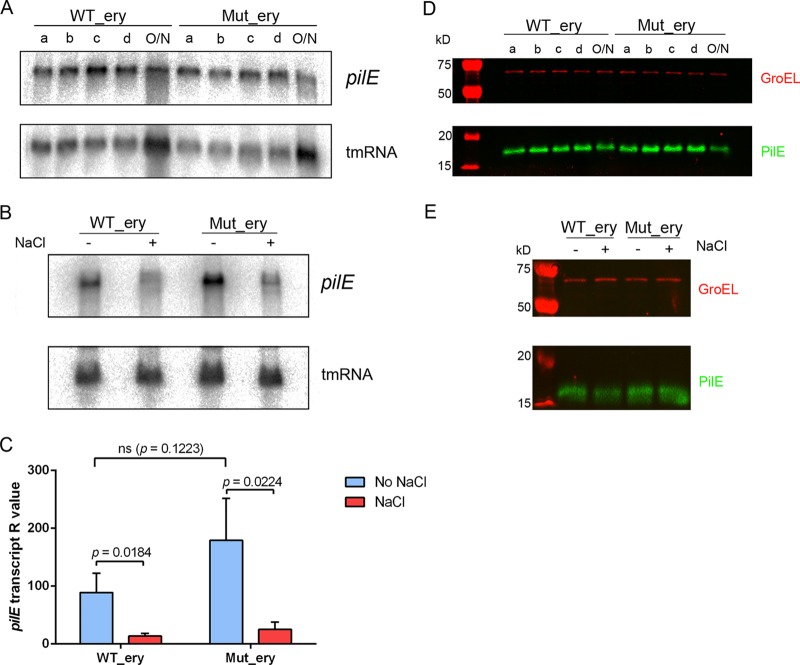
Effect of the AS RNA on *pilE* mRNA and PilE protein levels in N. meningitidis. (A) Northern blot analysis of RNA from WT_ery and Mut_ery taken at different time points during growth (shown in Fig. 3A). *pilE* transcript was detected using the *pilE*_probe. tmRNA was used as a loading control. (B) Detection of *pilE* transcript in N. meningitidis with and without NaCl stress. (C) Strand-specific qRT-PCR analysis of *pilE* mRNA levels in WT_ery and Mut_ery with and without NaCl stress. Average *R* values were calculated and analyzed as described in the text. The *pilE* transcript was downregulated under NaCl stress in both WT_ery and Mut_ery. (D) Western blot analysis of whole-cell lysates of WT_ery and Mut_ery taken at different time points during growth. GroEL was used as a loading control. (E) Western blot analysis results for WT_ery and Mut_ery with and without NaCl stress. GroEL was used as a loading control. Mutation of the AS promoter or NaCl stress did not result in a detectable difference in PilE protein levels.

Given that the AS RNA is complementary to the entire *pilE* coding sequence and 5′ UTR, we next determined whether *pilE* transcript or pilin protein levels are affected by the presence of the AS transcript. Northern blot analysis was performed using a *pilE*-specific oligonucleotide probe. Analysis of total RNA isolated from the strains at different growth phases revealed that *pilE* mRNA was expressed at similar levels throughout growth in both WT_ery and Mut_ery (Fig. 5A) and was detected even after overnight growth, when AS levels were highest (Fig. 3B). We also used NaCl stress to examine the levels of *pilE* transcript when expression of the AS RNA was induced. Total RNA was prepared from WT_ery and Mut_ery with and without brief incubation in high salt concentrations (0.5 M). We found that in the absence of NaCl stress, both Northern blot analysis and strand-specific qRT-PCR indicated a trend toward elevated *pilE* mRNA levels in Mut_ery compared to WT_ery (Fig. 5B and C; see also Table S1 in the supplemental material), but this was not significant (*P* = 0.1223; Student's *t* test). Following NaCl stress which induced AS RNA levels up to 280-fold (Fig. 3D), an approximately 5-fold reduction of *pilE* transcript levels was observed, but this was not dependent upon the AS transcript, as it was observed in both WT_ery and Mut_ery (*P* = 0.0184 and *P* = 0.0224, respectively; Student's *t* test).

Although there was no significant alteration in *pilE* transcript levels upon deletion or induction of the AS RNA, we investigated whether there were any changes in pilin protein levels, which might result from posttranscriptional regulation of PilE. We performed Western blot analysis of whole-cell lysates using anti-pilin antibody and fluorescence analysis to quantify relative amounts of pilin in whole-cell lysates from WT_ery and Mut_ery either during growth (Fig. 5D) or following salt stress (Fig. 5E). Mutation of the AS promoter or NaCl stress did not lead to a detectable change in PilE protein levels (for quantification, see Tables S3 and S4 in the supplemental material). Therefore, we found no evidence that the AS RNA affects *pilE* transcript or pilin protein levels in meningococcus.

### Effect of AS RNA on pilin antigenic variation.

A small G4-associated RNA is essential for pilin Av in N. gonorrhoeae (24). This small RNA is proposed to act in *cis*, forming an RNA:DNA hybrid with the C-rich DNA strand and promoting formation of the G4 structure to initiate gene conversion. Given that the AS RNA transcript overlaps with the promoter region of the G4-associated sRNA, we investigated whether the AS RNA had any impact on pilin Av.

**FIG 6:**
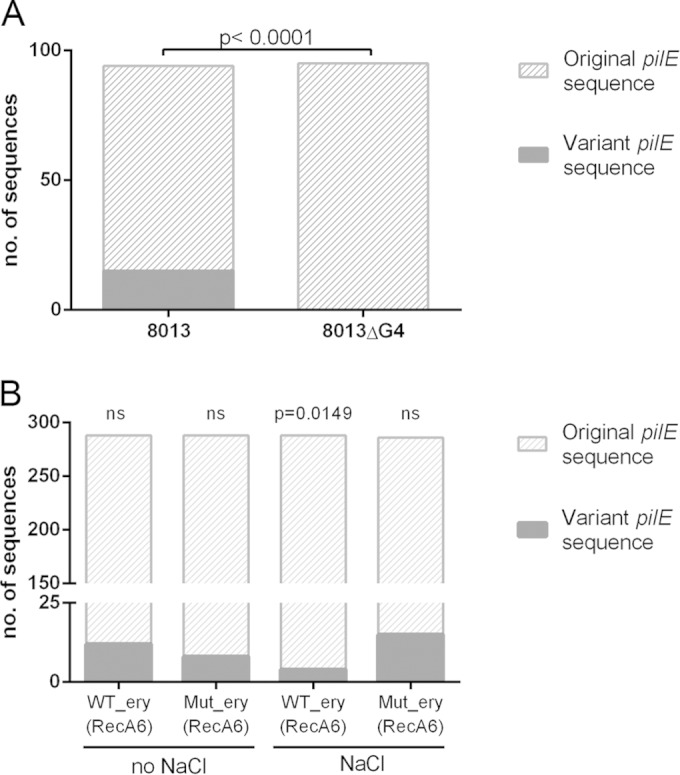
Effects on antigenic variation of AS mutation and AS induction under salt stress. (A) Av assays were performed with wild-type N. meningitidis strain 8013 and an isogenic mutant with a kanamycin resistance cassette inserted into the guanine quartet (G4) sequence. *pilE* sequences obtained were compared to the potential *pilS* donor sequences, and the number of variation events was calculated. No *pilE* variation events were detected in the strain with the G4 mutation. (B) Av assays were performed on WT_ery(RecA6) and Mut_ery(RecA6) with and without salt stress. Results were analyzed by fitting generalized linear models. NaCl stress resulted in a statistically significant reduction in antigenic variation in WT_ery(RecA6) (*P* = 0.0149) but not in Mut_ery(RecA6).

The G4 sequence and position relative to *pilE* is conserved in N. meningitidis strain 8013, and so we first performed Av assays using 8013 and 8013ΔG4, which has a kanamycin resistance marker with transcriptional terminators inserted into the G4 sequence (see Fig. S1 in the supplemental material). *pilE* variation was detected in 8013, while no Av events occurred in 8013ΔG4 (Fig. 6A), indicating that the G4 is required for Av in meningococcus and that Av is detectable by our methods. We therefore examined Av in WT_ery(RecA6) and Mut_ery(RecA6) in the presence of IPTG to induce RecA expression, with or without NaCl to induce the expression of the AS RNA. The sequence of *pilE* in the resulting colonies was compared to the potential *pilS* donor sequences in the N. meningitidis 8013 genome, and the number of variation events was determined. Table 3 and Fig. 6B show results from three independent assays (a total of 1,150 *pilE* sequences). We fitted a binomial GLH which proved to be satisfactory (goodness of fit statistic = 5.8392 on 6 degrees of freedom; *P* = 0.4414). On their own, neither the presence of NaCl nor the mutation resulted in a statistically significant change in levels of antigenic variation. However, we found that there was a significantly reduced level of antigenic variation when WT_ery was induced with NaCl (*z* = 2.434, *P* = 0.0149) but this was not observed for Mut_ery, indicating that the AS RNA transcript or transcription may influence pilin Av.

**TABLE 3:** *pilE* sequence variants detected in Av assays in liquid media

Strain/presence of NaCl	Assay replicate no.	Total no. of variants sequenced	No. of different *pilE* sequences	
			Per assay/strain	Per strain
WT_ery (RecA6)/−	1	96	5	12
	2	96	3	
	3	96	4	
Mut_ery (RecA6)/−	1	96	2	8
	2	96	3	
	3	96	3	
WT_ery (RecA6)/+	1	96	0	4
	2	96	1	
	3	96	3	
Mut_ery (RecA6)/+	1	96	7	15
	2	94	4	
	3	96	4	

## DISCUSSION

Here we have described the identification of a novel RNA antisense to *pilE* in N. meningitidis. The sequence and position of the promoter of this RNA are conserved in different meningococcal isolates expressing class I *pilE* but is absent from the class II *pilE* locus. Instead, the AS promoter is located in the *pilS* locus in class II-expressing strains, consistent with this region of the class II *pilS* locus having homology to the region 3′ of class I *pilE* (12). Analysis of the sequence downstream of *pilE* in two genomes of the related pathogen N. gonorrhoeae revealed conservation of the putative −35 sequence but substitutions in the putative −10 sequence. These base changes occur in the TATA box and thus may result in a nonfunctional promoter. Consistent with this, a recent transcriptomic study in N. gonorrhoeae did not detect antisense transcription in *pilE* (41); other reports have described the presence of functional antisense promoters in the 5′, 3′, and midgene regions of *pilE* in this species (S. Hill, T. Le, and S. Wachter, presented at the XIXth International Pathogenic Neisseria Conference, Ashville, NC, 12 to 17 October 2014). These diverse findings may reflect strain and species specificity; N. gonorrhoeae inhabits a physiologically distinct niche within human hosts compared to meningococcus and thus may employ different strategies for modulating *pilE* expression and variation. Additionally, changes to *pilE* CDS and/or 3′ UTR following gene conversion events could result in the introduction of promoter sequences within the *pilE* region. In strain 8013, we did not identify any additional AS promoters in or adjacent to any of the *pilS* cassettes, although we cannot exclude the presence of additional, midgene *pilE* AS promoters in other isolates of N. meningitidis. We note, however, that in an additional 200 genomes of meningococcal isolates from the United Kingdom publically available in the pubMLST database (http://pubmlst.org/neisseria/), the position and sequence of the *pilE* AS promoter identified in this work are conserved, despite variation in the *pilE* coding sequence (data not shown).

*cis*-encoded AS RNAs are abundant in bacteria (33), and 260 such RNAs were recently identified in a transcriptomic study of RNAs that are differentially regulated upon exposure of N. meningitidis to human blood (42). Interestingly, the *pilE* AS RNA was not identified in that study, consistent with our observation that the expression is regulated by specific conditions. We detected the AS transcript in late stationary phase or following a period of salt stress. Upregulation by salt was specific to NaCl and KCl, and not sucrose, implying the AS RNA is not induced by osmotic stress. The observation that salt also increases the promoter activity in E. coli indicates that the mechanisms may be common to both species, although the molecular basis of salt regulation of the AS RNA is not yet known. Bacterial sRNAs can be regulated in response to different environmental stresses. For example, the *trans*-encoded sRNAs DsrA and RprA, which are upregulated in E. coli by low-temperature and cell surface stress, respectively (43). In N. meningitidis the small RNA AniS is anaerobically induced (44), while NrrF is expressed by meningococcus under iron-depleted conditions (45).

*cis*-encoded AS RNAs have exact complementarity to the sense transcript and therefore often regulate the expression of the gene encoded on the opposite DNA strand. In most cases, this requires an RNA-RNA interaction and can result from transcription inhibition, alteration of transcript stability, or inhibition of translation (33). To date there has only been a single report of sRNA-mediated regulation of pili in bacteria. The sRNA FasX downregulates pilus expression in group A streptococci by targeting and destabilizing mRNA from the pilus biogenesis operon (46). Our work revealed that in N. meningitidis, *pilE* mRNA is detected throughout growth *in vitro* in both the wild type and the strain lacking the AS RNA, suggesting that the AS RNA is not required for stabilization of the *pilE* message. Furthermore, our results indicated that the *pilE* transcript is not destabilized by the AS RNA, as there was no significant AS RNA-dependent change in *pilE* transcript levels either in the mutant or when the AS was induced.

We considered that the AS RNA might alter pilin at the translational level. Our mapping experiments showed that the AS RNA includes the 5′ UTR of the *pilE* transcript. Noncoding RNAs can prevent translation by pairing with this region of mRNAs and affecting ribosome binding (47). Based on Western blotting, there was no difference in the amount of pilin in whole-cell lysates in the presence or absence of the AS RNA. Of note, however, we did observe that even when a decrease in the amount of *pilE* transcript was detected by Northern blotting following salt stress, there was no corresponding decrease in pilin protein levels detected in cell lysates. Upon expression, pilins are inserted into the inner membrane before cleavage by a prepilin peptidase and assembly into Tfp (48), which may constitute a stable pool of pilin. As such, further work is required to rule out any influence of AS RNA on pilin expression. For example, a sustained high level of AS RNA may impact the abundance of pilin. Consistent with this, piliation and pilin levels in N. gonorrhoeae are reduced during growth in 300 mM KCl or NaCl (49).

While our results do not provide any evidence for the AS RNA affecting *pilE* or pilin, this could be related to relative levels of the sense and AS transcripts. We estimate that the *pilE* transcript is over 200 times more abundant than the AS RNA, even when AS is induced by salt stress. Therefore, it is possible that the expression of *pilE* mRNA does in fact dictate AS RNA levels. Our data show that *pilE* mRNA is detected throughout growth, but when *pilE* transcript levels decrease (such as following salt stress or after overnight growth), the AS RNA levels increase. This increase may result from both increased AS promoter activity and the concomitant reduction of *pilE* transcription. Changes in the ratio of sense/AS transcripts, either in response to environmental cues or as governed by *pilE* transcript levels, could serve as a mechanism of regulation of AS functions.

The promoter of the G4-associated sRNA in N. gonorrhoeae is required for Av of *pilE* and has been proposed to result in transcription of an RNA that promotes G4 formation (24). In our assays, under AS-inducing conditions, we observed a significant decrease in *pilE* variation in the wild-type strain, which was not observed in the mutant. This suggested that, in contrast to the loss of the G4-associated sRNA which abolishes Av, AS RNA transcript or transcription modulates *pilE* variation. The mechanism by which this occurs is not yet known. Our mapping experiments revealed that the AS transcript 3′ end overlaps with the promoter of the G4-associated RNA. Overlapping transcription units can influence the expression of the downstream transcript, through promoter occlusion (50). Therefore, enhanced transcription of the AS RNA could interfere with the transcription of the G4-associated sRNA and consequently reduce the efficacy or frequency of G4 formation, and thus affect *pilE* variation frequency. Further work is under way to examine this possibility.

Like many noncoding RNAs, the AS RNA appears to be specifically expressed under certain conditions. As discussed, high concentrations of NaCl were used to induce AS RNA expression in this study. This level of salt is nonphysiological, although certain chronic inflammatory airway diseases, such as cystic fibrosis or rhinitis, have been reported to increase Na and Cl ion concentrations up to almost 300 mM in airway surface liquid (51). More importantly, we found that the AS RNA is expressed when bacteria are in stationary phase. This may resemble the environments that meningococci would encounter *in vivo*, for example competitive conditions of nutrient depletion, high cell density, and oxygen limitation (52). Little is known currently about the impact of the microenvironment on pilin expression and variation in the meningococcus. In N. gonorrhoeae, Av rates are not affected by temperature, carbon source, or oxygen availability, but an increase in Av frequency was observed when bacteria were grown under iron-starved conditions (53). Pilin variation is thought to be a key mechanism of immune escape, as well as impacting Tfp functions (5, 6, 14, 16, 17). Understanding the conditions which govern and modulate this sophisticated molecular process is therefore important. As is true for many noncoding RNAs, the precise role and mechanism of action of the AS RNA in Av remains elusive, but our work has identified a novel noncoding RNA in the *pilE* locus in N. meningitidis which influences Av in this important human pathogen. This study provides further insight into the complexity of pilin regulation and variation in pathogenic Neisseria spp.

## Supplementary Material

Supplemental material
