# Supplementary material for: Characterization of a Novel Antisense RNA in the Major Pilin Locus of Neisseria meningitidis Influencing Antigenic Variation
Source: J Bacteriol. 2015 Apr 17;197(10):1757–68. doi: 10.1128/JB.00082-15 (PMC4402397; doi:10.1128/JB.00082-15)
Supplement: Supplemental material [file supp_197_10_1757__index.html]

Supplemental material 

# Characterization of a Novel Antisense RNA in the Major Pilin Locus of Neisseria meningitidis Influencing Antigenic Variation

## Supplemental material

**Files in this Data Supplement:**

- Supplemental file 1 -

  Fig. S1 (Construction of strain 8014ΔG4), S2 (Antigenic variation assay), and S3 (AS RNA expression) and Tables S1 (AS transcript levels), S2 (*pilE* transcript levels), and S3 and S4 (Pilin protein levels)

  PDF, 395K
