# Supplementary material for: Characterization of a Novel Antisense RNA in the Major Pilin Locus of Neisseria meningitidis Influencing Antigenic Variation
Source: J Bacteriol. 2015 Apr 17;197(10):1757–68. doi: 10.1128/JB.00082-15 (PMC4402397; doi:10.1128/JB.00082-15)
Supplement: Supplemental material [file JB.00082-15_zjb999093601so1.pdf]

A

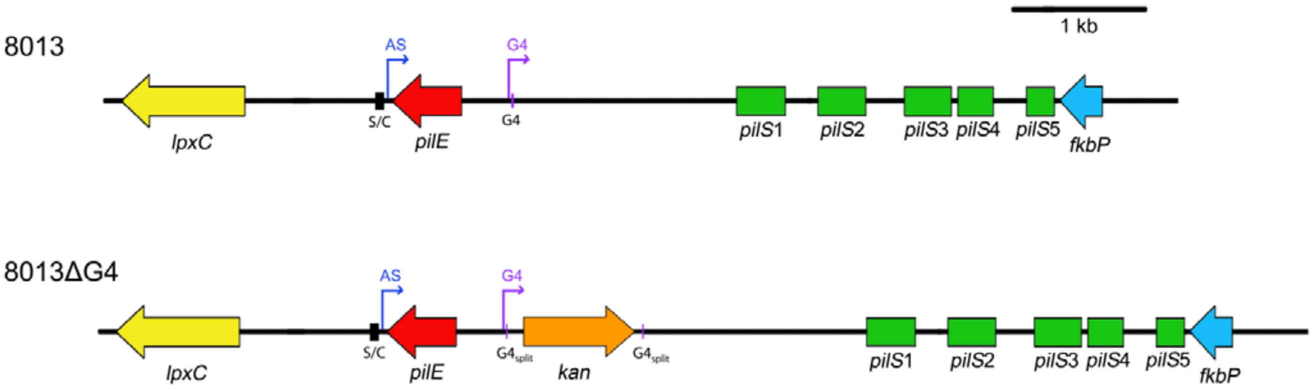

B

CATAAAATTACTCCTAATTGGAAAGGAAATGCCTCAAGCTTACGCCATCGGCATTATGCAATGTATTTGACCAT
CGGTATTTTGTTGCGATACCTGTGTATTATAAAGCAAGATTGGTATCAAGTTTGTGTTTTGAGGTGAAAATTTA
TGCGTTTATCTTTATGTGGTTGTTTTTATTTTACATTTTCTTTCGTTTGGGTGTGGTTTGAGGAAGCAGGGGAT
TACCGTTTTTTGTCAGCAGTGCCGAAAATTGTCAGTTTTAGTGCCGATTTTCGGCACTTTTTTATTGGCGTGGG
GTATCTCTATTGGCATGGGGCATCGGGTGTGTTGATTGGGTCGGAATTTGAGATTTTTGAATTTGCGCGGTAGC
ATA**GGGTGGG**ggatccCAACCATCATCGATGAATTGTGTCTCAAAATCTCTGATGTTACATTGCACAAGATA
AAAATATATCATCATGAACAATAAAACTGTCTGCTTACATAAACAGTAATACAAGGGGTGTTatgagccatatt
caacgggaaacgtcttgctcgaggccgcgattaaattccaacatggatgctgatttatatgggtataaatgggc
tcgcgataatgtcgggcaatcaggtgcgacaatctatcgattgtatgggaagcccgatgcgccagagttgtttc
tgaaacatggcaaaggtagcgttgccaatgatgttacagatgagatggtcagactaaactggctgacggaattt
atgcctcttccgaccatcaagcattttatccgtactcctgatgatgcatggttactcaccactgcgatccccgg
aaaaacagcattccaggtattagaagaatatcctgattcaggtgaaaatattgttgatgcgctggcagtgttcc
tgcgccggttgcattcgattcctgtttgtaattgtccttttaacagcgatcgcgtatttcgtctcgctcaggcg
caatcacgaatgaataacggtttggttgatgcgagtgattttgatgacgagcgtaatggctggcctgttgaaca
agtctggaaagaaatgcataaacttttgccattctcaccggattcagtcgtcactcatggtgatttctcacttg
ataaccttatttttgacgaggggaaattaataggttgtattgatgttggacgagtcggaatcgcagaccgatac
caggatcttgccatcctatggaactgcctcggtgagttttctccttcattacagaaacggctttttcaaaaata
tggtattgataatcctgatatgaataaattgcagtttcatttgatgctcgatgagtttttctaaTCAGAATTGG
TTAATTGGTTTGAGCTCATCAAATAAAACGAAAGGCTCAGTCGAAAGACTGGGCCTTTCGTTTTATCTGTTGAT
CAAATAAAACGAAAGGCTCAGTCGAAAGACTGGGCCTTTCGTTTTATCTGTTGGCATGCATCGggatcc**TTGG**
GTGGG

Figure S1: Schematic representation **(A)** and sequence **(B)** of the insertion of a kanamycin resistance cassette (orange) including two transcriptional terminators from the *E. coli* rrnB gene (underlined), into the Guanine quartet (G4) sequence of *N. meningitidis* strain 8013 (bold text) to construct strain 8014ΔG4. The putative G4 promoter -10 and -35 sequences are shown in underlined purple text. *Bam*HI restriction sites used for cloning purposes are in lower case.

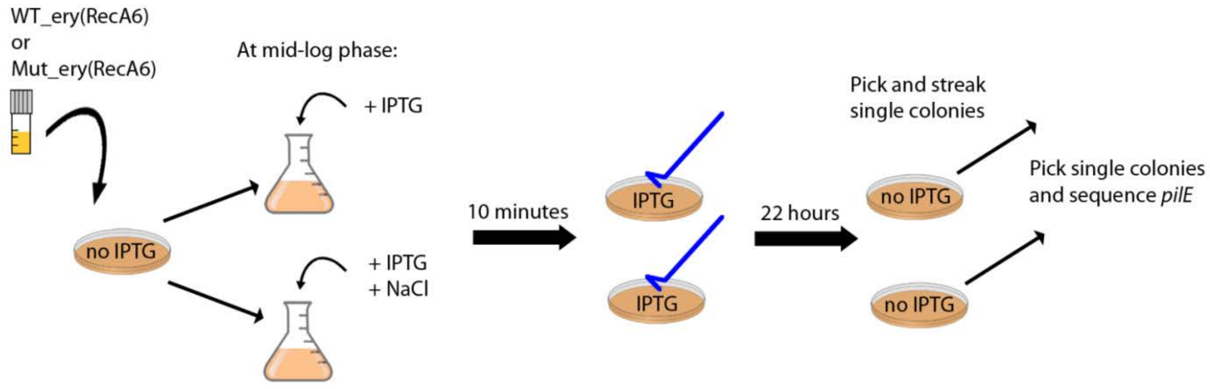

Figure S2: Schematic of Antigenic variation assay in liquid media. Strains WT_ery(RecA6) and Mut_ery(RecA6) were obtained by transformation of WT_ery or Mut_ery with gDNA from 8013(RecA6). The *pilE* sequence of single colonies of transformants was determined and frozen stocks of the strains were prepared. Strains grown overnight on solid BHI media were used to inoculate BHI broth and grown to mid-log phase. IPTG was added to a final concentration of 1 mM to allow expression of RecA and *pilE* variation. For NaCl stress, the cultures were incubated with NaCl (0.5 M) together with IPTG for 10 min. The cultures were then serially diluted and plated onto BHI agar containing IPTG and grown overnight for 22 hours. Ninety six colonies of each strain from each condition were passaged onto BHI agar and incubated overnight. *pilE* was amplified by PCR from single colonies, sequenced and compared to the sequence of the parent colony. For 8013 and 8013ΔG4 the same assay was performed but without IPTG or NaCl.

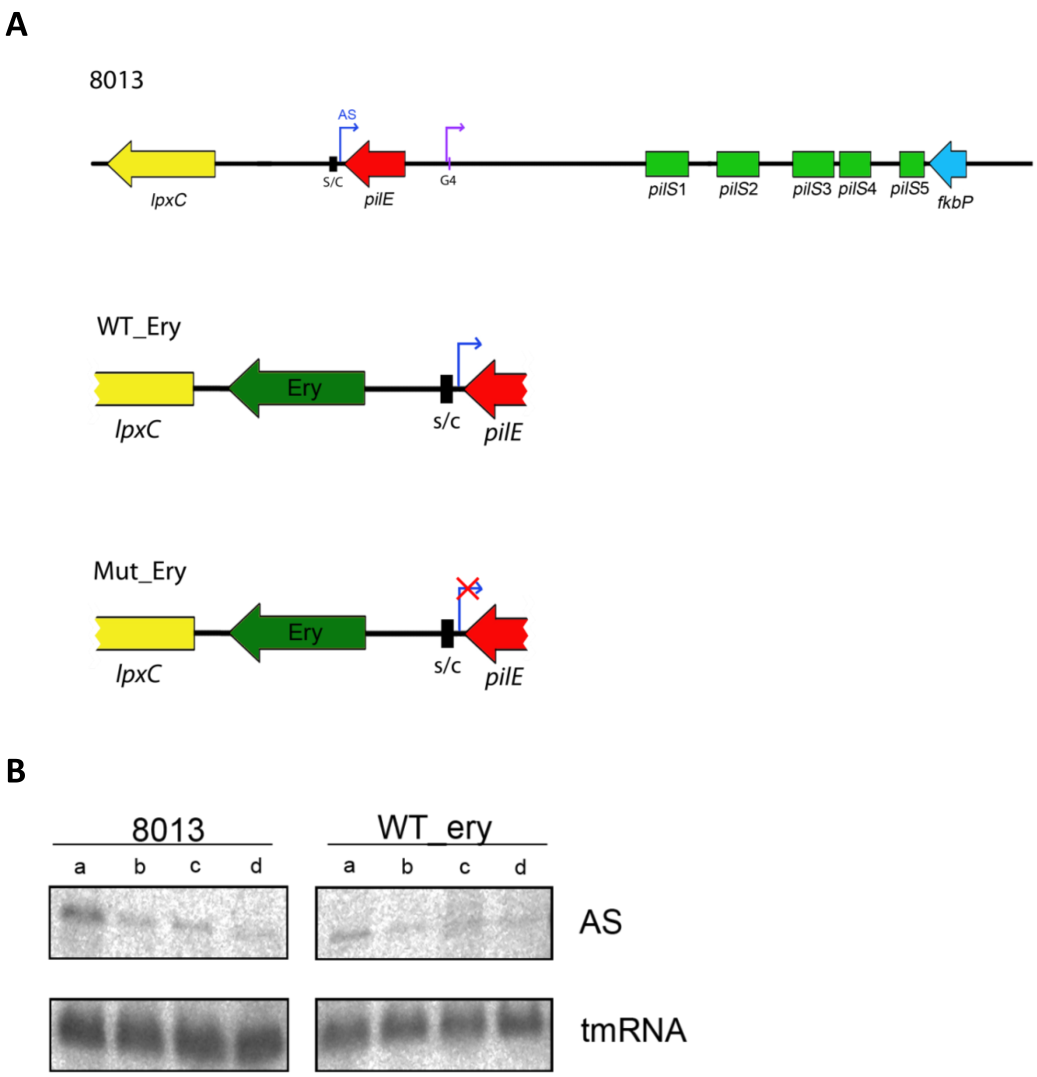

Figure S3: Expression of the AS RNA is not influenced by the presence of the erythromycin resistance cassette. (**A**) Schematic diagram of the *pilE* locus of 8013 wild-type strain and isogenic strains WT_ery and Mut_ery. The erythromycin resistance cassette comprising promoter and terminator sequences was inserted downstream of the *pilE* coding sequence and Sma/Cla repeat (S/C). G4 is the guanine quartet sequence required for pilin variation and the promoter for the G4 associated *cis*-encoded RNA promoter is indicated. (**B**) Comparison of AS RNA expression in wild type strain 8013 and WT_ery by Northern Blot analysis indicates that the resistance cassette does not impact AS RNA production. tmRNA was detected as a loading control.

TABLE S1. Quantification of AS transcript levels detected in Northern blots

Replicate	AS transcript			
	**WT_ery**		**Mut_ery**	
	No NaCl	NaCl	No NaCl	NaCl
1	1	42.043	0.554	0.032
2	1	540.078	0.294	0.916
3	1	324.92	0.099	0.284

Signals were detected using a fluorescent image analyzer (Fuji FLA-5000), and quantified using AIDA image analyzer software. Band intensities were normalised to the respective tmRNA band intensities and expressed as a ratio to the normalised value of the first lane.

Table S2. Quantification of *pilE* transcript levels detected in Northern blots.

**Replicate**	***pilE* transcript**			
	**WT_ery**		**Mut_ery**	
	No NaCl	NaCl	No NaCl	NaCl
1	1	0.117	1.658	0.218
2	1	0.216	1.573	0.166
3	1	0.234	1.85	0.292

Signals were detected using a fluorescent image analyzer (Fuji FLA-5000), and quantified using AIDA image analyzer software. Band intensities were normalised to the respective tmRNA band intensities and expressed as a ratio to the normalised value of the first lane.

Table S3. Quantification of pilin protein detected by Western blot at different time points during growth in liquid media.

	WT_ery					Mut_ery				
	a	b	c	d	O/N	a	b	c	d	O/N
PilE	1	1.312	1.124	1.575	0.968	1.188	1.743	1.773	1.713	0.840

Proteins were detected with fluorescently labelled antibodies and bands were visualised and quantified using Odyssey® Sa Infrared Imaging System. a-d correspond to different time points during growth as described in the main text. O/N is overnight growth. PilE band intensities were normalised to the respective GroEL band intensities and expressed as a ratio to the normalised PilE value of the first lane.

Table S4. Quantification of pilin protein detected by Western blot in WT_ery and Mut_ery with and without salt stress.

Replicate	PilE			
	WT_ery		MUT_ery	
	No NaCl	NaCl	No NaCl	NaCl
1	1	0.410	1.104	1.083
2	1	0.853	1.163	1.349
3	1	1.601	1.808	1.931

Proteins were detected with fluorescently labelled antibodies and bands were visualised and quantified using Odyssey® Sa Infrared Imaging System. Experiments were carried out in triplicate using strains from independent transformations. PilE band intensities were normalised to the respective GroEL band intensities and expressed as a ratio to the normalised PilE value of the first lane.
